# Crystal structure of tris­(1,3-dimesityl-4,5-di­hydro-1*H*-imidazol-3-ium) tetra­bromido­cobaltate(II) bromide chloro­form hexa­solvate

**DOI:** 10.1107/S2056989015016254

**Published:** 2015-09-12

**Authors:** Eduard Rais, Ulrich Flörke, René Wilhelm

**Affiliations:** a Department Chemie, Universität Paderborn, Warburgerstrasse 100, 33098 Paderborn, Germany

**Keywords:** crystal structure, imidazolium salt, absolute structure, C—H⋯Br inter­actions

## Abstract

In the unit cell of the title compound, (C_21_H_27_N_2_)_3_[CoBr_4_]Br·6CHCl_3_, the tetrabromidocobaltate(II) anion and the bromide anion are located on a crystallographic threefold rotation axis. For the [CoBr_4_]^2−^ group, the axis runs through one of the Br ligands and the Co^II^ atom. All other structure moieties lie on general sites. Various tris­(1,3-dimesityl-4,5-di­hydro-1*H*-imidazol-3-ium) structures with different counter-ions have been reported. In the title compound, the N—C—N angle is 113.7 (5)°, with short C—N bond lengths of 1.297 (7) and 1.307 (7) Å. The two mesityl planes make a dihedral angle of 34.6 (1)° and the dihedral angles between the mesityl and N–C–N planes are 82.0 (1) and 88.5 (1)°, respectively. The imidazoline ring is almost planar, with atom deviations in the range 0.003 (5)–0.017 (5) Å from the best plane; the mean deviation is 0.012 (5) Å. In the crystal, non-covalent inter­actions of the C—H⋯Br type occur between the Br^−^ anion and the cation, as well as between the [CoBr_4_]^2−^ anion and both the chloro­form solvent mol­ecules. These H⋯*A* distances are slightly shorter than the sum of van der Waals radii.

## Related literature   

For similar tris­(1,3-dimesityl-4,5-di­hydro-1*H*-imidazol-3-ium) structures, see: Arduengo *et al.* (1995 ▸); Hagos *et al.* (2008 ▸); Santoro *et al.* (2013 ▸); Buchalski *et al.* (2015 ▸). For synthesis of 2-bromo-1,3-dimesityl-4,5-di­hydro-1*H*-imidazol-3-ium bromide, see: Wiggins *et al.* (2012 ▸). For the application of 1,3-dimesityl-4,5-di­hydro-1*H*-imidazol-3-ium cation as a carbene precursor, see: Díez-González *et al.* (2009 ▸). For catalytic application of imidazolium based [CoCl_4_]^2−^ salts, see: Bica & Gärtner (2008 ▸); Wang *et al.* (2015 ▸).
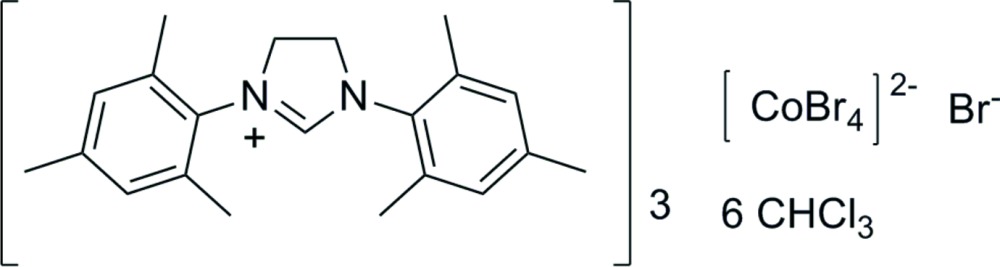


## Experimental   

### Crystal data  

(C_21_H_27_N_2_)_3_[CoBr_4_]Br·6CHCl_3_
*M* *_r_* = 2097.02
Trigonal, 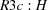

*a* = 16.0535 (14) Å
*c* = 61.790 (12) Å
*V* = 13791 (4) Å^3^
*Z* = 6
Mo *K*α radiation
μ = 2.92 mm^−1^
*T* = 130 K
0.22 × 0.21 × 0.20 mm

### Data collection  

Bruker SMART APEX diffractometer
Absorption correction: multi-scan (*SADABS*; Sheldrick, 2004 ▸) *T*_min_ = 0.279, *T*_max_ = 1.0
41734 measured reflections
7309 independent reflections
4427 reflections with *I* > 2σ(*I*)
*R*_int_ = 0.077

### Refinement  

*R*[*F* ^2^ > 2σ(*F* ^2^)] = 0.040
*wR*(*F* ^2^) = 0.078
*S* = 0.79
7309 reflections
306 parameters
1 restraint
H-atom parameters constrained
Δρ_max_ = 0.65 e Å^−3^
Δρ_min_ = −0.65 e Å^−3^
Absolute structure: Flack (1983 ▸), 1821 Friedel pairs
Absolute structure parameter: 0.020 (11)

### 

Data collection: *SMART* (Bruker, 2002 ▸); cell refinement: *SAINT* (Bruker, 2002 ▸); data reduction: *SAINT*; program(s) used to solve structure: *SHELXTL* (Sheldrick, 2008 ▸); program(s) used to refine structure: *SHELXL2013* (Sheldrick, 2015 ▸); molecular graphics: *SHELXTL*; software used to prepare material for publication: *SHELXTL* and local programs.

## Supplementary Material

Crystal structure: contains datablock(s) I, global. DOI: 10.1107/S2056989015016254/nr2061sup1.cif

Structure factors: contains datablock(s) I. DOI: 10.1107/S2056989015016254/nr2061Isup2.hkl

**Fig. 1.**
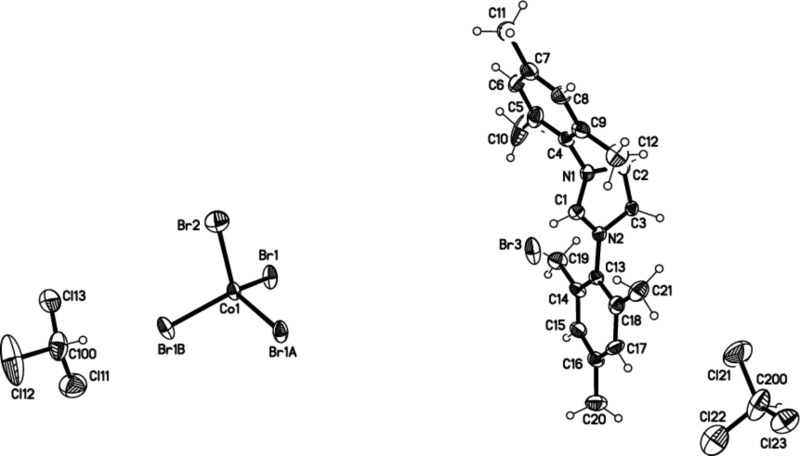
Molecular structure of the title compound with anisotropic displacement ellipsoids drawn at the 50% probability level. Non-stoichiometric representation.

**Fig. 2.**
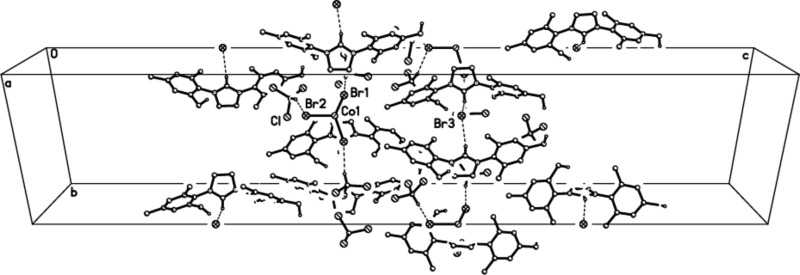
Crystal packing approximately viewed along a axis with intermolecular hydrogen bonding pattern drawn as dotted lines. H-atoms not involved are omitted.

. DOI: 10.1107/S2056989015016254/nr2061fig1.tif
Mol­ecular structure of the title compound with anisotropic displacement ellipsoids drawn at the 50% probability level. Non-stoichiometric representation.

a . DOI: 10.1107/S2056989015016254/nr2061fig2.tif
Crystal packing approximately viewed along *a* axis with inter­molecular hydrogen bonding pattern drawn as dotted lines. H-atoms not involved are omitted.

CCDC reference: 1421420

Additional supporting information:  crystallographic information; 3D view; checkCIF report

## Figures and Tables

**Table 1:** Hydrogen-bond geometry (, )

*D*H*A*	*D*H	H*A*	*D* *A*	*D*H*A*
C1H1*A*Br3^ii^	0.95	2.58	3.373(4)	141
C100H10Br1^i^	1.00	2.71	3.668(5)	161
C200H20Br2^iii^	1.00	2.54	3.454(6)	152

Symmetry codes: (i) 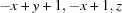; (ii) 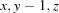; (iii) 
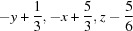
.

## supplementary crystallographic information

### S1. Synthesis and crystallization

All manipulations were carried out under nitro­gen atmosphere using standard Schlenk techniques. MeCN was dried with an MBRAUN MB SPS-800 solvent purification system under nitro­gen. CHCl_3_ was dried over activated molecular sieve with 3 Å pore diameter. 2-Bromo-1,3-dimesityl-4,5-di­hydro-1*H*-imidazol-3-ium bromide (0.080 g, 0.172 mmol, 1 eq) and Cobalt powder (0.227 g, 3.852 mmol, 22 eq, grain size < 150 µm) were filled into a sealed schlenk tube equipped with a stirring bar. MeCN (3 mL) was added and the mixture was stirred and heated at 100 °C for 40 h under inert gas. The cooled down mixture was filtrated and the solvent was then removed under vacuum. The residual brown oil was separated by decantation and was dissolved in CHCl_3_ (0.7 mL). Crystal growth could be observed after one day. The NMR spectra are not suitable because of the paramagnetic properties of the [CoBr_4_]^2-^ anion.

### S2. Refinement

Hydrogen atom positions were clearly derived from difference Fourier maps and then refined at idealized positions riding on the carbon atoms with isotropic displacement parameters U_iso_(H) = 1.2U(C_eq_) or 1.5U(-CH_3_) and C–H 0.95-1.00 Å. All CH_3_ hydrogen atoms were allowed to rotate but not to tip. It was not possible to refine a satisfactory split model for the C100–chloro­form.

### S3. Chemical Context

The cation is a common precursor for the synthesis of 1,3-dimesityl-4,5-di­hydro­imidazol-2-yl­idene, which is used as a ligand in various catalytic applications (Díez-González *et al.*, 2009). Alkyl-substituted imidazolium cations with [CoX_4_]^2-^ anions (X = Cl, Br) are used as metal-containing ionic liquids (Bica & Gaertner, 2008; Wang *et al.*, 2015).

### Crystal data

**Table:**

(C_21_H_27_N_2_)_3_[CoBr_4_]Br·6CHCl_3_	*D*_x_ = 1.515 Mg m^−^^3^
*M**_r_* = 2097.02	Mo *K*α radiation, λ = 0.71073 Å
Trigonal, *R*3*c*:*H*	Cell parameters from 4234 reflections
*a* = 16.0535 (14) Å	θ = 2.5–19.7°
*c* = 61.790 (12) Å	µ = 2.92 mm^−^^1^
*V* = 13791 (4) Å^3^	*T* = 130 K
*Z* = 6	Prism, pale-green
*F*(000) = 6306	0.22 × 0.21 × 0.20 mm

### Data collection

**Table:**

Bruker SMART APEX diffractometer	7309 independent reflections
Radiation source: sealed tube	4427 reflections with *I* > 2σ(*I*)
Graphite monochromator	*R*_int_ = 0.077
φ and ω scans	θ_max_ = 27.9°, θ_min_ = 1.6°
Absorption correction: multi-scan (*SADABS*; Sheldrick, 2004)	*h* = −21→21
*T*_min_ = 0.279, *T*_max_ = 1.0	*k* = −21→19
41734 measured reflections	*l* = −81→81

### Refinement

**Table:**

Refinement on *F*^2^	Secondary atom site location: difference Fourier map
Least-squares matrix: full	Hydrogen site location: difference Fourier map
*R*[*F*^2^ > 2σ(*F*^2^)] = 0.040	H-atom parameters constrained
*wR*(*F*^2^) = 0.078	*w* = 1/[σ^2^(*F*_o_^2^) + (0.0298*P*)^2^] where *P* = (*F*_o_^2^ + 2*F*_c_^2^)/3
*S* = 0.79	(Δ/σ)_max_ = 0.001
7309 reflections	Δρ_max_ = 0.65 e Å^−^^3^
306 parameters	Δρ_min_ = −0.64 e Å^−^^3^
1 restraint	Absolute structure: Flack (1983), 1821 Friedel pairs
Primary atom site location: structure-invariant direct methods	Absolute structure parameter: 0.020 (11)

### Special details

**Table:**

Geometry. All e.s.d.'s (except the e.s.d. in the dihedral angle between two l.s. planes) are estimated using the full covariance matrix. The cell e.s.d.'s are taken into account individually in the estimation of e.s.d.'s in distances, angles and torsion angles; correlations between e.s.d.'s in cell parameters are only used when they are defined by crystal symmetry. An approximate (isotropic) treatment of cell e.s.d.'s is used for estimating e.s.d.'s involving l.s. planes.

### Fractional atomic coordinates and isotropic or equivalent isotropic displacement parameters (Å^2^)

**Table:**

	*x*	*y*	*z*	*U*_iso_*/*U*_eq_	
Co1	0.6667	0.3333	0.91225 (2)	0.0290 (3)	
Br1	0.77035 (4)	0.49648 (5)	0.92389 (2)	0.04336 (19)	
Br2	0.6667	0.3333	0.87362 (2)	0.0630 (4)	
Br3	0.3333	0.6667	0.91594 (3)	0.0524 (4)	
N1	0.4682 (3)	−0.0461 (3)	0.90279 (8)	0.0296 (12)	
N2	0.4612 (3)	−0.0455 (3)	0.93802 (8)	0.0281 (12)	
C1	0.4610 (4)	−0.0918 (4)	0.92081 (10)	0.0292 (14)	
H1A	0.4561	−0.1533	0.9213	0.035*	
C2	0.4754 (4)	0.0476 (4)	0.90750 (10)	0.0338 (16)	
H2A	0.4225	0.0526	0.9005	0.041*	
H2B	0.5378	0.1017	0.9026	0.041*	
C3	0.4669 (4)	0.0462 (4)	0.93224 (9)	0.0270 (14)	
H3A	0.5239	0.1015	0.9389	0.032*	
H3B	0.4082	0.0472	0.9368	0.032*	
C4	0.4725 (4)	−0.0782 (4)	0.88091 (10)	0.0319 (15)	
C5	0.5614 (5)	−0.0511 (5)	0.87196 (13)	0.049 (2)	
C6	0.5625 (6)	−0.0800 (5)	0.85046 (12)	0.055 (2)	
H6A	0.6222	−0.0621	0.8437	0.065*	
C7	0.4769 (7)	−0.1346 (6)	0.83901 (12)	0.054 (2)	
C8	0.3921 (6)	−0.1592 (5)	0.84898 (11)	0.0464 (18)	
H8A	0.3340	−0.1964	0.8412	0.056*	
C9	0.3867 (5)	−0.1327 (4)	0.86980 (11)	0.0375 (16)	
C10	0.6534 (5)	0.0048 (6)	0.88517 (16)	0.087 (3)	
H10A	0.6525	−0.0346	0.8974	0.130*	
H10B	0.6577	0.0641	0.8907	0.130*	
H10C	0.7092	0.0210	0.8760	0.130*	
C11	0.4845 (7)	−0.1602 (6)	0.81586 (12)	0.080 (3)	
H11A	0.5414	−0.1673	0.8143	0.120*	
H11B	0.4902	−0.1090	0.8062	0.120*	
H11C	0.4268	−0.2209	0.8120	0.120*	
C12	0.2910 (5)	−0.1610 (5)	0.87956 (12)	0.0503 (19)	
H12A	0.2405	−0.1947	0.8687	0.075*	
H12B	0.2902	−0.1032	0.8844	0.075*	
H12C	0.2795	−0.2035	0.8919	0.075*	
C13	0.4495 (4)	−0.0797 (4)	0.96013 (10)	0.0293 (14)	
C14	0.5308 (4)	−0.0439 (4)	0.97348 (11)	0.0356 (16)	
C15	0.5164 (5)	−0.0718 (4)	0.99472 (11)	0.0395 (17)	
H15A	0.5707	−0.0479	1.0040	0.047*	
C16	0.4255 (5)	−0.1339 (4)	1.00332 (11)	0.0403 (17)	
C17	0.3468 (5)	−0.1667 (5)	0.98966 (12)	0.0454 (19)	
H17A	0.2845	−0.2091	0.9953	0.055*	
C18	0.3559 (5)	−0.1394 (4)	0.96770 (12)	0.0378 (16)	
C19	0.6300 (4)	0.0195 (5)	0.96462 (11)	0.0487 (19)	
H19A	0.6332	0.0760	0.9577	0.073*	
H19B	0.6447	−0.0163	0.9539	0.073*	
H19C	0.6769	0.0403	0.9764	0.073*	
C20	0.4143 (6)	−0.1603 (5)	1.02691 (12)	0.059 (2)	
H20A	0.4242	−0.2153	1.0290	0.089*	
H20B	0.3495	−0.1774	1.0317	0.089*	
H20C	0.4620	−0.1054	1.0354	0.089*	
C21	0.2699 (4)	−0.1763 (5)	0.95317 (12)	0.0503 (19)	
H21A	0.2725	−0.2195	0.9423	0.076*	
H21B	0.2698	−0.1221	0.9459	0.076*	
H21C	0.2111	−0.2116	0.9618	0.076*	
C100	0.8893 (6)	0.1327 (6)	0.92865 (13)	0.070 (3)	
H10	0.8362	0.1455	0.9248	0.084*	
Cl11	0.91290 (19)	0.1546 (2)	0.95548 (4)	0.0938 (8)	
Cl12	0.8521 (2)	0.0149 (2)	0.92250 (7)	0.1562 (18)	
Cl13	0.9911 (3)	0.2121 (2)	0.91398 (5)	0.1293 (12)	
C200	0.0704 (6)	0.8507 (6)	0.01629 (18)	0.091 (3)	
H20	0.0749	0.9066	0.0245	0.109*	
Cl21	0.1365 (2)	0.8900 (2)	−0.00698 (6)	0.1410 (14)	
Cl22	0.11836 (19)	0.7912 (2)	0.03238 (5)	0.1032 (9)	
Cl23	−0.05036 (16)	0.76626 (15)	0.01124 (4)	0.0790 (7)	

### Atomic displacement parameters (Å^2^)

**Table:**

	*U*^11^	*U*^22^	*U*^33^	*U*^12^	*U*^13^	*U*^23^
Co1	0.0214 (5)	0.0214 (5)	0.0440 (10)	0.0107 (2)	0.000	0.000
Br1	0.0253 (3)	0.0267 (3)	0.0769 (5)	0.0121 (3)	−0.0062 (4)	−0.0129 (3)
Br2	0.0687 (6)	0.0687 (6)	0.0515 (9)	0.0344 (3)	0.000	0.000
Br3	0.0263 (4)	0.0263 (4)	0.1045 (11)	0.01314 (19)	0.000	0.000
N1	0.030 (3)	0.027 (3)	0.033 (3)	0.015 (2)	−0.001 (2)	−0.004 (2)
N2	0.032 (3)	0.026 (3)	0.028 (3)	0.016 (2)	0.003 (2)	0.000 (2)
C1	0.023 (3)	0.024 (3)	0.034 (4)	0.007 (3)	0.002 (3)	0.002 (3)
C2	0.032 (4)	0.025 (3)	0.046 (5)	0.015 (3)	−0.004 (3)	−0.004 (3)
C3	0.031 (3)	0.027 (3)	0.027 (4)	0.017 (3)	−0.001 (3)	−0.005 (3)
C4	0.041 (4)	0.024 (3)	0.031 (4)	0.016 (3)	0.004 (3)	0.004 (3)
C5	0.051 (5)	0.034 (4)	0.065 (6)	0.023 (4)	0.013 (4)	−0.001 (4)
C6	0.072 (6)	0.049 (5)	0.056 (6)	0.041 (4)	0.036 (5)	0.017 (4)
C7	0.092 (6)	0.056 (5)	0.037 (5)	0.055 (5)	−0.005 (5)	−0.002 (4)
C8	0.062 (5)	0.056 (5)	0.029 (4)	0.034 (4)	−0.001 (4)	−0.007 (4)
C9	0.052 (4)	0.032 (4)	0.031 (4)	0.023 (3)	−0.005 (3)	−0.003 (3)
C10	0.029 (4)	0.098 (7)	0.125 (9)	0.026 (5)	0.007 (5)	−0.044 (6)
C11	0.149 (9)	0.094 (7)	0.044 (6)	0.095 (7)	0.010 (5)	0.005 (5)
C12	0.043 (4)	0.047 (4)	0.050 (5)	0.014 (4)	−0.009 (4)	−0.015 (4)
C13	0.037 (4)	0.020 (3)	0.032 (4)	0.015 (3)	0.004 (3)	0.002 (3)
C14	0.039 (4)	0.027 (3)	0.039 (5)	0.015 (3)	0.001 (3)	0.010 (3)
C15	0.047 (4)	0.040 (4)	0.035 (5)	0.024 (4)	0.000 (3)	0.007 (3)
C16	0.060 (5)	0.034 (4)	0.034 (4)	0.030 (4)	0.005 (4)	0.004 (3)
C17	0.052 (5)	0.036 (4)	0.045 (5)	0.021 (4)	0.028 (4)	0.011 (4)
C18	0.041 (4)	0.027 (4)	0.046 (5)	0.017 (3)	0.005 (3)	−0.005 (3)
C19	0.034 (4)	0.050 (4)	0.050 (5)	0.012 (4)	−0.001 (3)	0.007 (4)
C20	0.073 (6)	0.052 (5)	0.052 (5)	0.030 (4)	0.019 (4)	0.021 (4)
C21	0.035 (4)	0.050 (4)	0.058 (5)	0.015 (4)	0.011 (4)	0.000 (4)
C100	0.076 (6)	0.086 (6)	0.074 (7)	0.061 (6)	−0.032 (5)	−0.030 (5)
Cl11	0.106 (2)	0.119 (2)	0.0673 (17)	0.0651 (18)	−0.0027 (14)	0.0048 (15)
Cl12	0.159 (3)	0.107 (2)	0.248 (5)	0.101 (2)	−0.115 (3)	−0.096 (3)
Cl13	0.192 (3)	0.165 (3)	0.115 (2)	0.153 (3)	0.073 (2)	0.067 (2)
C200	0.061 (6)	0.049 (5)	0.147 (10)	0.016 (5)	0.028 (6)	−0.021 (6)
Cl21	0.128 (3)	0.114 (2)	0.181 (4)	0.060 (2)	0.098 (3)	0.067 (2)
Cl22	0.096 (2)	0.0933 (19)	0.107 (2)	0.0379 (16)	−0.0029 (17)	−0.0331 (17)
Cl23	0.0694 (15)	0.0597 (14)	0.0825 (17)	0.0131 (12)	0.0122 (13)	−0.0093 (12)

### Geometric parameters (Å, º)

**Table:**

Co1—Br2	2.387 (2)	C11—H11C	0.9800
Co1—Br1	2.4057 (8)	C12—H12A	0.9800
Co1—Br1^i^	2.4057 (8)	C12—H12B	0.9800
Co1—Br1^ii^	2.4057 (8)	C12—H12C	0.9800
N1—C1	1.307 (7)	C13—C18	1.397 (8)
N1—C4	1.460 (7)	C13—C14	1.402 (8)
N1—C2	1.479 (7)	C14—C15	1.368 (8)
N2—C1	1.297 (7)	C14—C19	1.500 (8)
N2—C13	1.449 (7)	C15—C16	1.397 (9)
N2—C3	1.472 (7)	C15—H15A	0.9500
C1—H1A	0.9500	C16—C17	1.386 (10)
C2—C3	1.534 (8)	C16—C20	1.504 (9)
C2—H2A	0.9900	C17—C18	1.411 (9)
C2—H2B	0.9900	C17—H17A	0.9500
C3—H3A	0.9900	C18—C21	1.499 (9)
C3—H3B	0.9900	C19—H19A	0.9800
C4—C5	1.383 (9)	C19—H19B	0.9800
C4—C9	1.389 (8)	C19—H19C	0.9800
C5—C6	1.410 (10)	C20—H20A	0.9800
C5—C10	1.525 (10)	C20—H20B	0.9800
C6—C7	1.397 (10)	C20—H20C	0.9800
C6—H6A	0.9500	C21—H21A	0.9800
C7—C8	1.360 (10)	C21—H21B	0.9800
C7—C11	1.510 (10)	C21—H21C	0.9800
C8—C9	1.371 (9)	C100—Cl11	1.698 (8)
C8—H8A	0.9500	C100—Cl12	1.716 (8)
C9—C12	1.494 (9)	C100—Cl13	1.741 (9)
C10—H10A	0.9800	C100—H10	1.0000
C10—H10B	0.9800	C200—Cl21	1.709 (10)
C10—H10C	0.9800	C200—Cl23	1.751 (9)
C11—H11A	0.9800	C200—Cl22	1.797 (11)
C11—H11B	0.9800	C200—H20	1.0000
			
Br2—Co1—Br1	107.39 (4)	H11B—C11—H11C	109.5
Br2—Co1—Br1^i^	107.39 (4)	C9—C12—H12A	109.5
Br1—Co1—Br1^i^	111.47 (4)	C9—C12—H12B	109.5
Br2—Co1—Br1^ii^	107.39 (4)	H12A—C12—H12B	109.5
Br1—Co1—Br1^ii^	111.47 (4)	C9—C12—H12C	109.5
Br1^i^—Co1—Br1^ii^	111.47 (4)	H12A—C12—H12C	109.5
C1—N1—C4	126.6 (5)	H12B—C12—H12C	109.5
C1—N1—C2	110.1 (5)	C18—C13—C14	123.0 (6)
C4—N1—C2	123.2 (5)	C18—C13—N2	117.9 (5)
C1—N2—C13	126.4 (5)	C14—C13—N2	118.8 (5)
C1—N2—C3	110.8 (5)	C15—C14—C13	117.4 (6)
C13—N2—C3	122.6 (5)	C15—C14—C19	121.4 (6)
N2—C1—N1	113.7 (5)	C13—C14—C19	121.2 (6)
N2—C1—H1A	123.1	C14—C15—C16	123.0 (6)
N1—C1—H1A	123.1	C14—C15—H15A	118.5
N1—C2—C3	102.7 (5)	C16—C15—H15A	118.5
N1—C2—H2A	111.2	C17—C16—C15	117.9 (6)
C3—C2—H2A	111.2	C17—C16—C20	121.7 (6)
N1—C2—H2B	111.2	C15—C16—C20	120.3 (7)
C3—C2—H2B	111.2	C16—C17—C18	122.3 (6)
H2A—C2—H2B	109.1	C16—C17—H17A	118.9
N2—C3—C2	102.5 (4)	C18—C17—H17A	118.9
N2—C3—H3A	111.3	C13—C18—C17	116.4 (6)
C2—C3—H3A	111.3	C13—C18—C21	122.2 (6)
N2—C3—H3B	111.3	C17—C18—C21	121.4 (6)
C2—C3—H3B	111.3	C14—C19—H19A	109.5
H3A—C3—H3B	109.2	C14—C19—H19B	109.5
C5—C4—C9	122.9 (6)	H19A—C19—H19B	109.5
C5—C4—N1	118.9 (6)	C14—C19—H19C	109.5
C9—C4—N1	118.2 (5)	H19A—C19—H19C	109.5
C4—C5—C6	117.0 (7)	H19B—C19—H19C	109.5
C4—C5—C10	121.0 (7)	C16—C20—H20A	109.5
C6—C5—C10	122.0 (7)	C16—C20—H20B	109.5
C7—C6—C5	120.9 (7)	H20A—C20—H20B	109.5
C7—C6—H6A	119.5	C16—C20—H20C	109.5
C5—C6—H6A	119.5	H20A—C20—H20C	109.5
C8—C7—C6	118.7 (7)	H20B—C20—H20C	109.5
C8—C7—C11	123.7 (8)	C18—C21—H21A	109.5
C6—C7—C11	117.5 (8)	C18—C21—H21B	109.5
C7—C8—C9	123.0 (7)	H21A—C21—H21B	109.5
C7—C8—H8A	118.5	C18—C21—H21C	109.5
C9—C8—H8A	118.5	H21A—C21—H21C	109.5
C8—C9—C4	117.6 (6)	H21B—C21—H21C	109.5
C8—C9—C12	119.9 (6)	Cl11—C100—Cl12	111.3 (5)
C4—C9—C12	122.5 (6)	Cl11—C100—Cl13	109.0 (5)
C5—C10—H10A	109.5	Cl12—C100—Cl13	112.0 (5)
C5—C10—H10B	109.5	Cl11—C100—H10	108.1
H10A—C10—H10B	109.5	Cl12—C100—H10	108.1
C5—C10—H10C	109.5	Cl13—C100—H10	108.1
H10A—C10—H10C	109.5	Cl21—C200—Cl23	112.2 (6)
H10B—C10—H10C	109.5	Cl21—C200—Cl22	108.1 (5)
C7—C11—H11A	109.5	Cl23—C200—Cl22	106.8 (5)
C7—C11—H11B	109.5	Cl21—C200—H20	109.9
H11A—C11—H11B	109.5	Cl23—C200—H20	109.9
C7—C11—H11C	109.5	Cl22—C200—H20	109.9
H11A—C11—H11C	109.5		
			
C13—N2—C1—N1	176.7 (5)	C5—C4—C9—C8	−0.7 (9)
C3—N2—C1—N1	1.7 (7)	N1—C4—C9—C8	177.6 (5)
C4—N1—C1—N2	178.3 (5)	C5—C4—C9—C12	−179.5 (6)
C2—N1—C1—N2	0.4 (7)	N1—C4—C9—C12	−1.2 (9)
C1—N1—C2—C3	−2.2 (6)	C1—N2—C13—C18	−82.7 (7)
C4—N1—C2—C3	179.9 (5)	C3—N2—C13—C18	91.8 (7)
C1—N2—C3—C2	−2.9 (6)	C1—N2—C13—C14	103.6 (7)
C13—N2—C3—C2	−178.1 (5)	C3—N2—C13—C14	−81.9 (7)
N1—C2—C3—N2	2.8 (5)	C18—C13—C14—C15	1.8 (9)
C1—N1—C4—C5	−91.4 (7)	N2—C13—C14—C15	175.0 (5)
C2—N1—C4—C5	86.2 (7)	C18—C13—C14—C19	179.6 (6)
C1—N1—C4—C9	90.3 (7)	N2—C13—C14—C19	−7.1 (9)
C2—N1—C4—C9	−92.1 (7)	C13—C14—C15—C16	0.5 (9)
C9—C4—C5—C6	1.1 (9)	C19—C14—C15—C16	−177.3 (6)
N1—C4—C5—C6	−177.2 (5)	C14—C15—C16—C17	−1.3 (10)
C9—C4—C5—C10	−177.1 (7)	C14—C15—C16—C20	−178.8 (6)
N1—C4—C5—C10	4.6 (10)	C15—C16—C17—C18	−0.1 (9)
C4—C5—C6—C7	−0.8 (10)	C20—C16—C17—C18	177.3 (6)
C10—C5—C6—C7	177.3 (7)	C14—C13—C18—C17	−3.0 (9)
C5—C6—C7—C8	0.3 (10)	N2—C13—C18—C17	−176.4 (5)
C5—C6—C7—C11	178.3 (6)	C14—C13—C18—C21	179.7 (6)
C6—C7—C8—C9	0.1 (11)	N2—C13—C18—C21	6.3 (9)
C11—C7—C8—C9	−177.8 (6)	C16—C17—C18—C13	2.2 (9)
C7—C8—C9—C4	0.1 (10)	C16—C17—C18—C21	179.5 (6)
C7—C8—C9—C12	178.9 (6)		

Symmetry codes: (i) −*x*+*y*+1, −*x*+1, *z*; (ii) −*y*+1, *x*−*y*, *z*.

### Hydrogen-bond geometry (Å, º)

**Table:**

*D*—H···*A*	*D*—H	H···*A*	*D*···*A*	*D*—H···*A*
C1—H1*A*···Br3^iii^	0.95	2.58	3.373 (4)	141
C100—H10···Br1^i^	1.00	2.71	3.668 (5)	161
C200—H20···Br2^iv^	1.00	2.54	3.454 (6)	152

Symmetry codes: (i) −*x*+*y*+1, −*x*+1, *z*; (iii) *x*, *y*−1, *z*; (iv) −*y*+1/3, −*x*+5/3, *z*−5/6.
