# Supplementary figures and images for: Crystal structure of tris­(1,3-dimesityl-4,5-di­hydro-1H-imidazol-3-ium) tetra­bromido­cobaltate(II) bromide chloro­form hexa­solvate
Source: Acta Crystallogr E Crystallogr Commun. 2015 Sep 12;71(Pt 10):m177–8. doi: 10.1107/S2056989015016254 (PMC4647430; doi:10.1107/S2056989015016254)

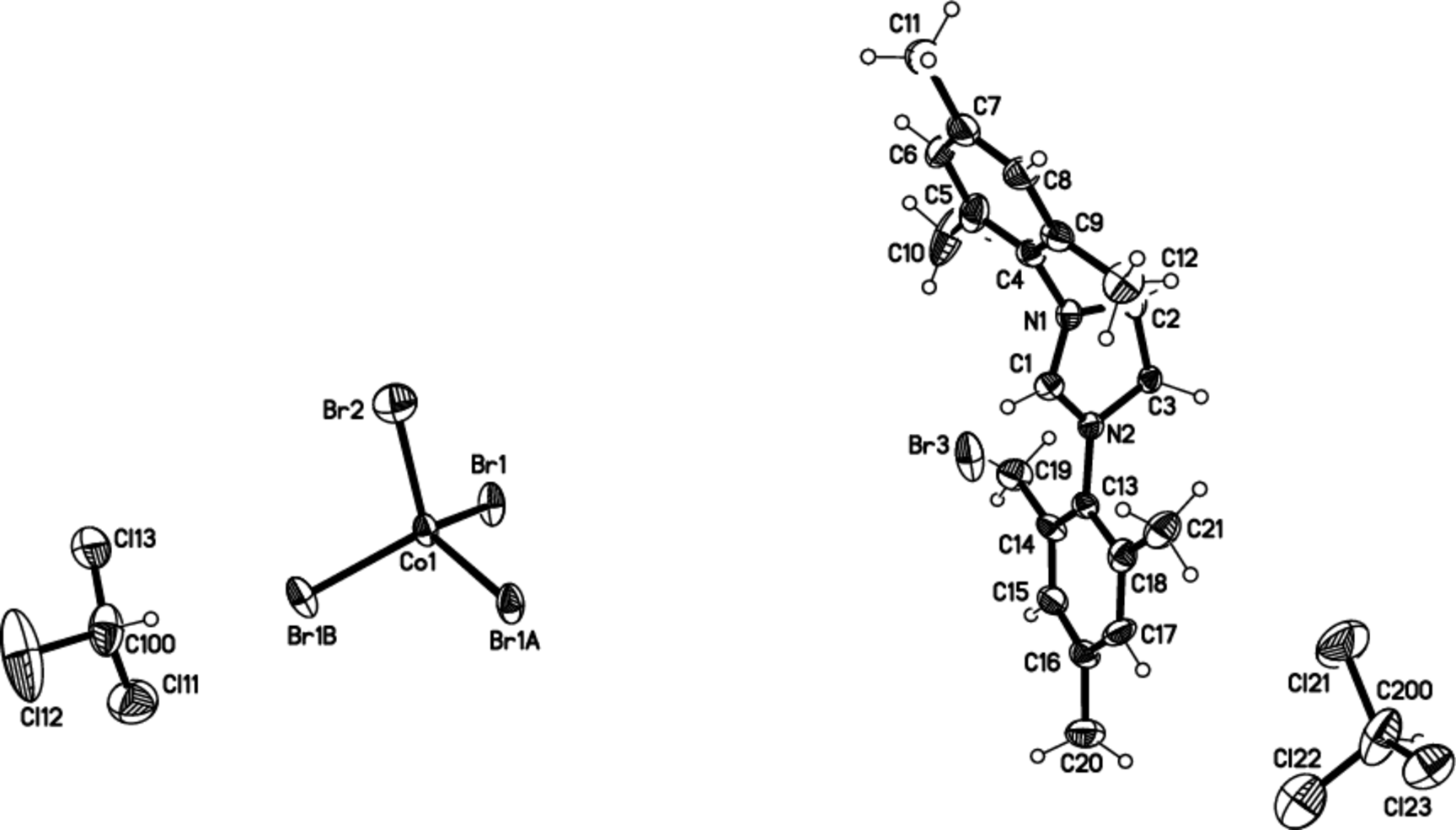

Supplement: Supplementary file 3 [file e-71-0m177-fig1.tif]

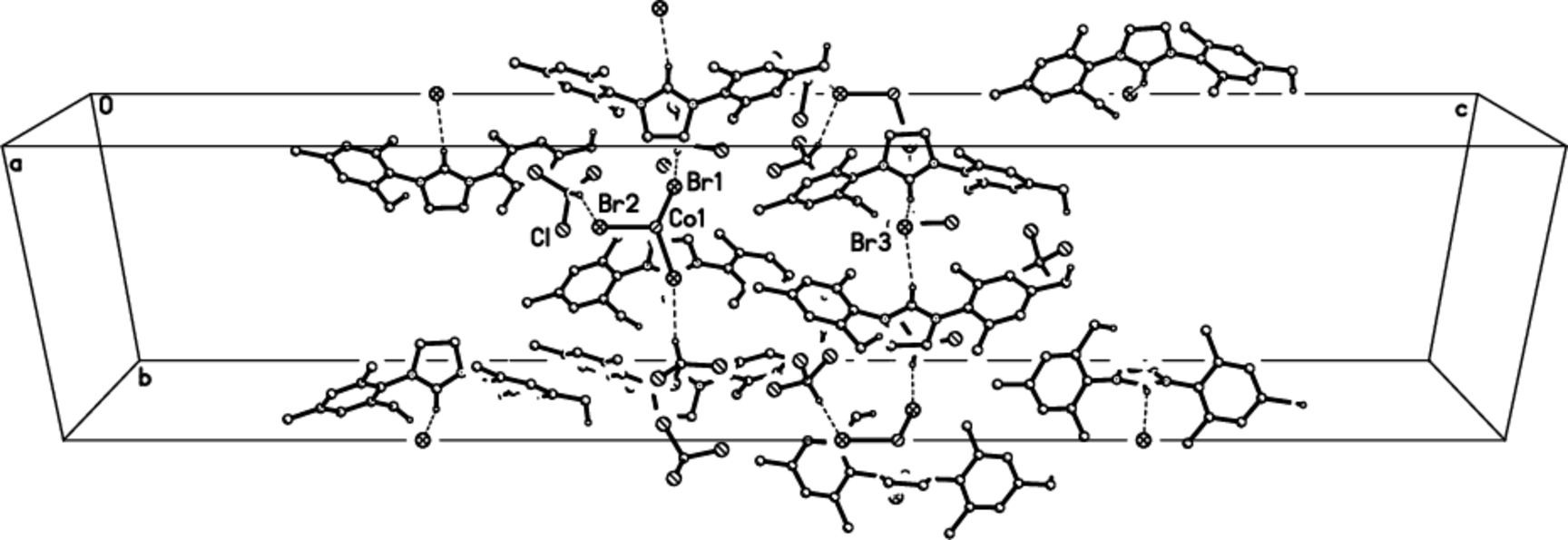

Supplement: Supplementary file 4 [file e-71-0m177-fig2.tif]
